# Nephrons and non-relapse mortality: simplified comorbidity index and acute kidney injury are associated with NRM in adults undergoing allogeneic hematopoietic cell transplant

**DOI:** 10.3389/frtra.2024.1352413

**Published:** 2024-03-18

**Authors:** Clark Raymond Robinson, Alma Habib, Nattawat Klomjit, Qing Cao, Shernan Grace Holtan

**Affiliations:** ^1^ Division of Hematology and Oncology, University of Texas Southwestern Medical Center, Dallas, TX, United States; ^2^ Department of Hematology, The Ohio State University, Columbus, OH, United States; ^3^ Division of Nephrology and Hypertension, University of Minnesota, Minneapolis, MN, United States; ^4^ Biostatistics and Bioinformatics, Masonic Cancer Center, University of Minnesota, Minneapolis, MN, United States; ^5^ Division of Hematology, Oncology, and Transplantation, University of Minnesota, Minneapolis, MN, United States

**Keywords:** non-relapse mortality, simplified comorbidity index, chronic kidney disease, acute kidney injury, allogeneic hematopoietic stem cell transplant

## Abstract

The Simplified Comorbidity Index (SCI) is a recently published 5-component, pre-transplant tool to predict non-relapse mortality (NRM) in allogeneic hematopoietic cell transplantation (alloHCT) patients. The SCI captures chronic kidney disease (CKD) using estimated glomerular filtration rate (eGFR) based on the CKD-EPI equation (KDIGO 2021 CKD-EPI), which may be more sensitive to predict risk of NRM than the creatinine cut-off in the 16-component, Hematopoietic Cell Transplant—Comorbidity Index (HCT-CI). We retrospectively assessed the ability of the SCI to risk-stratify patients and the impact of acute kidney injury (AKI) to NRM in adults who underwent alloHCT at the University of Minnesota. We included 373 patients who underwent their first alloHCT between 2015 and 2019. Through multivariate analysis, we found that patients with an SCI of greater than 4 had a higher risk of NRM. Additionally, we noted that AKIs stages 2–3 prior to day +100 was independently associated with a 3-fold greater NRM than patients who did not experience clinically significant AKI.

## 1 Introduction

Hematopoietic stem cells from a healthy donor can be transplanted into a patient with a hematologic malignancy or bone marrow disorder through a procedure known as a hematopoietic cell transplantation. Rapid restoration of donor-derived hematopoiesis is essential for the success of the procedure (1, 2). Additionally, the new donor-derived immune system provides immunologic control over the underlying disease, often termed the graft-vs.-tumor effect. While the transplant primarily involves hematopoietic cells developing into a new immune system, the process can potentially lead to toxicities in any organ system, with the kidneys being particularly susceptible. Because of these toxicities, physicians specializing in hematopoietic stem cell transplantation use pre-transplant risk assessment tools to guide which patients would be able to tolerate undergoing a hematopoietic stem cell transplant.

The Hematopoietic Cell Transplant—Comorbidity Index (HCT-CI) consists of 15 components and is the standard tool for assessing pre-transplant risk of non-relapse mortality (NRM) in patients undergoing an evaluation for an allogeneic hematopoietic cell transplant (alloHCT) (3). The Simplified Comorbidity Index (SCI) is a recently published alternative to the HCT-CI, developed by Shouval et al. that scores patients on a scale from 0 to 12 (with a score of 12 in patients with the highest risk of NRM) based on 5 components (age, cardiac dysfunction, pulmonary dysfunction, hepatic dysfunction, and renal dysfunction) (4). The SCI may be a simpler and more accurate tool for assessing NRM in patients being considered for alloHCT, with the main distinguishing feature between the two scores being the method of categorization of renal function. One difference between the SCI and HCT-CI is that the SCI assesses renal dysfunction based on the patient's estimated glomerular filtration rate (eGFR), whereas the HCT-CI uses a creatinine cutoff of >2.0 mg/dl. Thus, we sought to assess the impact of SCI on post-transplant survival using the SCI. Furthermore, recognizing the important role of renal function in withstanding regimen-related toxicities, we investigated the impact of AKI on post-transplant outcomes.

## 2 Methods

The study was approved by the University of Minnesota (UMN) institutional board of review, STUDY00010473. We included adult patients (age ≥18 years) who underwent their first alloHCT at the UMN between January 2015 and December 2019 (*N* = 373). We retrospectively calculated the SCI scores for adults and assessed the association of SCI scores with NRM, overall survival (OS), relapse, development of AKI, need for intensive care unit stay, length of initial hospitalization, need for dialysis, and incidence of acute and chronic graft-vs.-host disease (GVHD). AKI was defined by the Kidney Disease Improving Global Outcomes (KDIGO) 2012 guideline (5). We utilized the 2021 non-race based eGFR equation to reflect the current practice (6).

### 2.1 Statistical analysis

Continuous variables were summarized by median and range whereas categorical variables were summarized by value and percentage. Survival was estimated using Kaplan-Meier, with differences in curves estimated by log-rank tests. Multivariate analysis for NRM was performed using a Cox model adjusted for age, HCT-CI, SCI, conditioning intensity, and development of grade II-IV acute GVHD, and stage 0–1 vs. 2–3 AKI within the first 100 days of alloHCT. *P*-value <0.05 was considered statistically significant. An area under the receiver operator curve was done to compare NRM estimates of the SCI vs. HCT-CI. We categorized our cohort in to four categories based on conditioning regimens and donor-recipient histocompatibility [i.e., matched myeloablative conditioning (MAC), mismatched MAC, matched non-myeloablative/reduced intensity condition (NMA/RIC), mismatched NMA/RIC] where “matched” configurations were defined as six out of six human leukocyte antigen (HLA) matches between the donor and recipient; eight of eight HLA matches; and six out of six, plus six out of six HLA matches for recipients of double umbilical cord donor transplants. Statistical analyses were completed using JMP Pro 17 (SAS Corporation, Cary, NC). Figures were created using GraphPad Prism version 9.4.1 (GraphPad Software, Boston, MA).

## 3 Results

### 3.1 Demographics

Patient baseline characteristics are detailed in Table 1. Our cohort was 38% female with a median age of 56 years (range 18–75 years) and 89% receiving calcineurin inhibitor-based GVHD prophylaxis.

**Table 1:** Patient baseline characteristics.

Variable	SCI < 4 (*N* = 349)	SCI 4+ (*N* = 24)
Age (median, range, year)	55 (18–75)	65.5 (49–72)
Sex (*N*, %)		
Male	211 (60%)	19 (79%)
Female	138 (40%)	5 (21%)
Disease (*N*, %)		
AML	138 (40%)	9 (37%)
MDS	52 (15%)	6 (25%)
ALL	57 (16%)	0 (0%)
Lymphoma	37 (11%)	4 (17%)
Other	63 (18%)	5 (21%)
Graft source (*N*, %)		
Marrow	106 (30%)	10 (42%)
PBSC	137 (40%)	10 (42%)
UCB	106 (30%)	4 (16%)
Donor match (*N*, %)		
Matched sibling	135 (39%)	13 (54%)
Matched URD	53 (15%)	3 (13%)
Mismatched URD	4 (1%)	0 (0%)
Haploidentical	51 (15%)	4 (16%)
Single UCB	59 (17%)	1 (4%)
Double UCB	47 (13%)	3 (13%)
Conditioning regimen (*N*, %)		
RIC/NMA	213 (61%)	22 (92%)
MAC	136 (39%)	2 (8%)
HCT-CI (*N*, %)		
0	88 (25%)	0 (0%)
1–2	136 (39%)	7 (29%)
3+	123 (35%)	17 (71%)
Not available	2 (1%)	0 (0%)
GVHD Prophylaxis (*N*, %)		
CNI/MMF +/− others	184 (53%)	18 (75%)
CNI/MTX +/− others	45 (13%)	2 (8%)
Sirolimus/MMF	37 (10%)	1 (4%)
PTCy/Tac/MMF	81 (23%)	3 (13%)
T-cell depletion	2 (1%)	0 (0%)
Baseline eGFR (median, range)	105 (65–127)	81.5 (50–104.5)
AKI by stage (*N* = 373)		
Stage 0 (*N* = 49, 13%)	46 (13%)	3 (12%)
Stage 1 (*N* = 122, 33%)	111 (33%)	11 (46%)
Stage 2 (*N* = 134, 36%)	129 (37%)	5 (21%)
Stage 3 (*N* = 68, 18%)	63 (18%)	5 (21%)

AML, acute myeloid leukemia; ALL, acute lymphocytic leukemia; CNI, calcineurin inhibitors; eGFR, estimated glomerular filtration rate; GVHD, graft vs. host disease; HCT-CI, hematopoietic cell transplant comorbidity index; MAC, myeloablative conditioning; MMF, mycophenolate mofetil; MTX, methotrexate; NMA, non-myeloablative conditioning; PBSC, peripheral blood stem cells; PTCy, post-transplant cyclophosphamide; RIC, reduced intensity conditioning; SCI, simplified comorbidity index; UCB, umbilical cord blood; URD, unrelated donor.

### 3.2 SCI score impact outcome of AlloHCT

**Figure 1:**
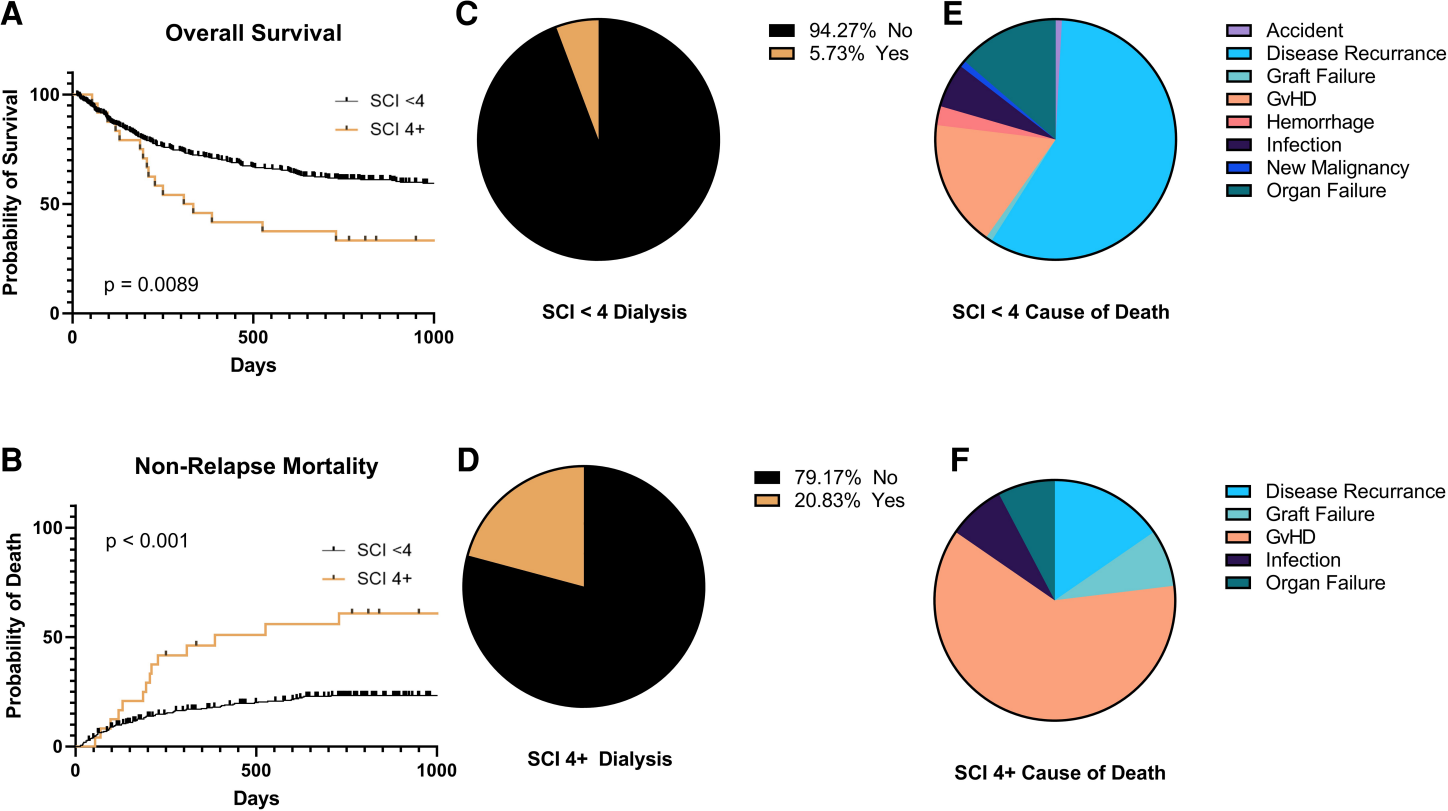
Clinical outcomes by simplified comorbidity Index (SCI) <4 or 4 + . (**A**) Overall survival, (**B**) non-relapse mortality, (**C**,**D**) need for hemodialysis in the first 100 days post-transplant, and (**E**,**F**) causes of death.

There was no statistically significant difference in survival between SCI scores 0–3 (*p* = 0.1). Therefore, we dichotomized the SCI variable into two groups: SCI <4 and ≥4, with a median OS of 1,549 days vs. 321 days, (*p* = 0.0089, Figure 1A) with a 2-year NRM of 23% and 61%, respectively (*p* < 0.001, Figure 1B). The need for ICU care (14% vs. 25%, *p* = 0.16) and median length of hospital stay (27 vs. 23 days, *p* = 0.25) were similar between those with a low vs. high SCI score. Moderate to severe AKI (AKI stage 2 or above) was not more common in SCI ≥4 compared to SCI <4 (*p* = 0.2). Moderate/severe AKI post-transplant was associated with a baseline eGFR of <75 ml/min/1.73 m^2^ (55% vs. 29% for those with higher baseline eGFR, *p* = 0.045). However, patients with an SCI of ≥4 were also more likely to need dialysis post-transplant (21% vs. 5.3%, *p* = 0.02, Figures 1C,D). SCI was not associated with the development of acute or chronic GVHD (*p* = 0.12 and *p* = 0.24, respectively) or relapse (*p* = 0.66). However, patients with an SCI of 4 or greater had an excess mortality due to GVHD (61.5% of deaths) vs. those with a lower SCI (17.1% of deaths, *p* = 0.04, Figures 1E,F). In multivariate analysis, an SCI score of ≥4 and stage 2–3 AKI before day +100 were both independently associated with worse 2-year NRM (Table 2).

**Table 2:** Multivariate model for non-relapse mortality.

Variable	Hazard ratio	Lower 95%	Upper 95%	*P*-value
AKI				
0–1	Reference			
2–3	3.1	1.9	5.1	<0.001
SCI				
<4	Reference			
4+	2.5	1.3	4.7	0.005
Age				
<65	Reference			
65+	2.3	1.3	3.8	0.002
AGVHD II-IV				
No	Reference			
Yes	1.9	1.2	3.0	0.003
Conditioning				
RIC/NMA	Reference			
MAC	1.1	0.6	1.9	0.8
HCT-CI				
0	Reference			
1–2	1.5	0.8	2.9	0.2
3+	1.5	0.8	2.8	0.3

AGVHD, acute GVHD; AKI, acute kidney injury; HCT-CI, hematopoietic cell transplant comorbidity index; MAC, myeloablative conditioning; NMA, non-myeloablative conditioning; RIC, reduced intensity conditioning; SCI, simplified comorbidity index.

### 3.3 Area under the curve analysis

We observed a significant difference between the area under the ROC for NRM at one year (0.59 vs. 0.42, *p*-value = 0.02) and 2 years (0.56 vs. 0.44, *p*-value = 0.046) between SCI and HCT-CI in the NMA/RIC matched population. No significant differences were observed for matched MAC, mismatched MAC, and mismatched NMA/RIC groups (Supplementary Table S1).

## 4 Discussion

In our cohort, we found that patients with higher SCI particularly greater than 4 had the highest risk of NRM. We also noted that moderate to severe AKI (AKI stage 2–3) prior to day +100 was associated with higher NRM. Risk stratification remains an important part of medical decision making and treatment planning in alloHCT. Only a small number of studies have looked at the SCI and evaluated clinical outcomes based upon this simplified risk stratification system. Shouval et al. retrospectively assigned an internal and an external cohort into SCI subgroups of patients with SCI scores of 1, 2, 3, 4+, and found that increasing SCI scores directly correlated with increasing NRM. Elias et al. also demonstrated greater NRM with increasing SCI in patients who underwent NMA/RIC and received allografts from matched donors (7). Unlike Shouval et al. and Elias et al., however, we did not see increasing NRM with increasing SCI within individual strata, but we did see a clear increase in NRM for patients with SCI scores of 4 and greater. Therefore, we dichotomized our data into SCI groups <4 and ≥4, as an SCI of 4 was the most relevant threshold for NRM risk within our dataset (see Supplementary Materials).

Several differences between the aforementioned cohorts and our study population could help explain these differences in results. Shouval's cohort received MAC with CD34-selected allografts that did not require post-transplant calcineurin inhibitors (CNIs), whereas our study population was a mixed population of MAC and NMA/RIC that mostly received post-transplant CNIs. CNIs are well known to increase the risk of AKI in the post-transplant setting (8). AKIs have been independently shown to increase NRM in alloHCT patients by others (9). Our current analysis supports the need to avoid post-transplant AKI, as we found the occurrence of AKIs greater than stage 2 within the first 100 days was associated with an increased NRM.

Elias's cohort only consisted of patients who underwent NMA/RIC and received allografts from matched donors, whereas our cohort of mixed conditioning regimens also varied by the source of their allografts (i.e., matched-related, matched unrelated, haploidentical, single umbilical cord blood and double umbilical cord blood). Haploidentical allografts (10) and umbilical cord blood allografts (11) carry risks for mortality greater than with matched donors, which could explain the discordance in Elias's results vs. our own.

A comparison of Shouval's and Elias's univariate analyses supports the notion that differences in conditioning regimens and allografts can change the weight of the components of the SCI. For example, Shouval et al. found that all pulmonary dysfunction strongly correlated with increasing NRM, whereas Elias only saw a significant correlation of increasing NRM with severe pulmonary dysfunction. In addition, Shouval et al. found moderate-severe hepatic dysfunction in MAC correlated with increasing NRM, but Elias did not see a significant correlation with hepatic dysfunction in their NMA/RIC patients.

These differences in condition regimens and graft sources between the studied populations are likely confounders between the findings of Shouval and Elias and our study, as we applied the SCI to a general population to assess the generalizability of the scoring system. In support of this notion, we demonstrated better discrimination of the SCI over the HCT-CI when we subcategorized our population into four groups based on their conditioning regimens and donor-recipient histocompatibility (i.e.,) and performed an area under the receiver operator curve (ROC) analysis for NRM comparing SCI vs. HCT-CI. We found a significant difference in NRM between SCI and HCT-CI for our NMA/RIC matched group at 1 and 2 years. We did not find a difference in our other groups. However, the NMA/RIC matched group was our largest subcategory, and the lack of difference in our other groups may be due to insufficient power to detect statistical differences. Even within the NMA/RIC matched group, the AUCs were still fair to poor at <0.7, suggesting a need to continue to improve methods of NRM risk estimation. Further studies with larger cohorts may better answer these questions.

Regardless of these differences between the various cohorts, Shouval, Elias and our study all demonstrated that pre-transplant renal dysfunction (as measured by eGFR) was one of the strongest predictors of NRM. For example, Shouval demonstrated that patient's with eGFRs of 60–90 ml/min/1.73 m^2^ and eGFRs of <60 ml/min/1.73 m^2^ had NRM rates of 35.8% and 57.5%, respectively. The SCI's use of eGFR over the creatinine cutoff used by the HCT-CI may be a more sensitive and accurate indicator of renal dysfunction, as creatinine is directly related to a patient's muscle mass. Patients with sarcopenia may have a creatinine less than 2.0 mg/dl, but still have significant renal dysfunction that is not captured by the HCT-CI (12).

This finding raises the question of whether PTCy use in patients with baseline renal comorbidity could be a way to avoid CNI exposure as PTCy lessens the need for post-transplant immunosuppression with CNI, with emerging results of substituting sirolimus showing promise (13). Our institution transitioned to PTCy for most adult alloHCT procedures as its method for prophylaxis against GVHD between 2018 and 2021. We are currently investigating the incidence of AKI in PTCy patients vs. patients who received other methods of GVHD prophylaxis, and longer follow-up is needed. Irrespective of CNI use, the aggregate data appears clear that baseline pre-transplant renal dysfunction increases the inherent risks of alloHCT, as patients may have less renal resilience to withstand AKIs related to infections, dehydration, and medications.

Fludarabine and cyclophosphamide are conditioning agents that are renally cleared and associated with toxicities at supratherapeutic levels (14, 15). Interestingly, Shadman et al. described the experiences of six patients who successfully underwent alloHCT while on renal replacement therapy (16). These patients had their doses of fludarabine and cyclophosphamide renally-dosed, and their levels were monitored, suggesting possible approaches for mitigating the risks of alloHCT in patients with advanced chronic kidney disease.

Shouval et al. note that the HCT-CI was developed from a retrospective analysis at from 1997 to 2003, but transplantation practices have significantly changed over the past two decades, highlighting the need for an updated tool for assessing pre-transplant NRM in alloHCT patients. Furthermore, Shouval et al. point out that the HCT-CI has varying validity across centers and cohorts, and in their multivariable analysis of each component of the HCT-CI, only pulmonary disease and moderate to severe hepatic dysfunction were significantly correlated to NRM in their cohort. Supporting this statement, SCI's area under the curve (AUC) for NRM compared to HCT-CI was higher at all time points with the largest difference at the 1-year mark.

The current landscape of pre-transplant risk assessment tools consists of the HCT-CI, Disease Risk Index (DRI), the the European Group for Bone Marrow Transplant (EBMT) risk score, the Comprehensive Geriatric Assessment (CGA), and the Pretransplant Assessment Mortality (PAM). DRI is only applicable to candidates undergoing a transplant for malignant reasons and only considers the nature of the disease, but does not account for the patient's individual pre-transplant health conditions that might affect their outcomes. Additionally, the DRI may lack data on newly identified prognostic molecular markers that may affect outcomes as more targeted therapies for specific mutations are developed (17). The EBMT risk score was developed in 1998 for chronic myeloid leukemia (CML) patients and requires the modification and validation of the EBMT score for non-CML patients. Furthermore, the EBMT has an age cutoff of 40 years as it was developed at a time when MAC was not offered to patients over the age of 50, limiting its applicability in the era of NMA/RIC and less strict age cutoffs (18). The HCT-CI can be cumbersome to calculate with 15 different components leading to significant interobserver disagreements in scoring without proper training in the scoring system (19). The CGA is a tool that may be most useful in geriatric patients being assessed for alloHCT. Unfortunately, the CGA is time-consuming to calculate as it includes a battery of physical and cognitive exams and its applicability is limited to an older population. Additionally, the CGA is not a stand-alone tool and was developed as a supplemental tool to the HCT-CI (20). The PAM is easier to calculate with only 8 components, but like the HCT-CI, uses a strict creatinine cut-off, whereas the eGFR cut-off of the SCI may be a more accurate measure of a candidate's renal function and their pre-transplant risk (21).

In conclusion, the SCI offers an attractive alternative to the HCT-CI as a pre-transplant assessment tool in allograft patients due to its greater simplicity and accuracy than the HCT-CI. The SCI comprises 5 variables, while the HCT-CI includes 15 variables. More importantly, Shouval and Elias found in their multivariate analysis that many of the components of HCT-CI were not associated with risk in more modern data sets, and the clinically significant components of the SCI had a higher AUC for NRM than the HCT-CI in their respective selected populations, suggesting a more accurate risk assessment tool. Taken together with recent analyses, we show that renal function is a critical risk factor for NRM in alloHCT, not adequately captured by the HCT-CI, and suggest that more attention be devoted to assess and mitigate renal disease in alloHCT patients before and after transplant.

## Data Availability

The original contributions presented in the study are included in the article/**Supplementary Material**, further inquiries can be directed to the corresponding author.
