# Supplementary material for: Nephrons and non-relapse mortality: simplified comorbidity index and acute kidney injury are associated with NRM in adults undergoing allogeneic hematopoietic cell transplant
Source: Front Transplant. 2024 Mar 18;3:1352413. doi: 10.3389/frtra.2024.1352413 (PMC11235361; doi:10.3389/frtra.2024.1352413)
Supplement: Supplementary file 1 [file Datasheet1.docx]

Supplementary Material

# Supplementary Figures and Tables

## Supplementary Figures

**Supplemental Figure 1. Overall Survival by raw SCI score.**


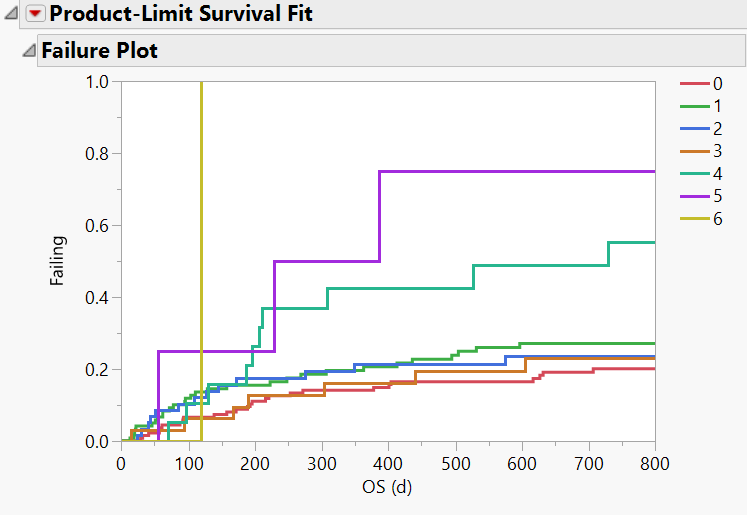


**Supplemental Table 1. Table Models of NRM discrimination by the area under the receiver operating curve (ROC).**

| **Time**    **(Months from alloHCT) Model** |  | **12 months** | | | **24 months** | | |
| --- | --- | --- | --- | --- | --- | --- | --- |
|  | **N** | **SCI** | **HCT-CI** | **p-value** | **SCI** | **HCT-CI** | **p-value** |
| **MA Matched** |  |  |  |  |  |  |  |
| NRM AUC(%)  [95%] | 84 | 37.76  [25.47, 50.05] | 43.41  [26.62, 60.21] | 0.35 | 41.68  [27.37, 55.99] | 37.16  [21.34, 52.98] | 0.40 |
| **MA Mismatched** |  |  |  |  |  |  |  |
| NRM AUC(%)  [95%] | 54 | 59.94  [43.44, 76.44] | 58.77  [41.86, 75.68] | 0.85 | 62.63  [47.71, 77.55] | 64.78  [49.43, 80.13] | 0.72 |
| **RIC Matched** |  |  |  |  |  |  |  |
| NRM AUC(%)  [95%] | 139 | 59.03  [46.21, 71.85] | 42.84  [31.02, 54.66] | 0.02 | 56.36  [44.44, 68.28] | 43.68  [33.15, 54.21 | 0.046 |
| **RIC Mismatched** |  |  |  |  |  |  |  |
| NRM AUC(%)  [95%] | 95 | 54.98  [39.9, 70.03] | 49.13  [34.06, 64.20] | 0.54 | 57.43  [43.75, 71.11 | 52.69  [38.60, 66.78] | 0.59 |
